# Quality Evaluation of *Dendrobium huoshanense* Under Different Cultivation Modes and Its Protective Effect on Ethanol-Induced Injury in GES-1 Cells

**DOI:** 10.3390/plants14243718

**Published:** 2025-12-05

**Authors:** Xinxin Li, Ziting Zhu, Jing Wu, Nianjun Yu, Huiqun Xie, Lan Han, Daiyin Peng

**Affiliations:** 1 College of Pharmacy, Anhui University of Chinese Medicine, Hefei 230012, China; lixinxin20000316@163.com (X.L.); zhuziting2024@163.com (Z.Z.); wuqingzheng55@163.com (J.W.); nianjunyu@ahtcm.edu.cn (N.Y.); xhq121342@ahtcm.edu.cn (H.X.); 2 Ministry of Education-Anhui Joint Collaborative Innovation Center for Quality Improvement of Anhui Genuine Chinese Medicinal Materials, Hefei 230012, China; 3 Institute of Traditional Chinese Medicine Resources Protection and Development, Anhui Academy of Chinese Medicine, Hefei 230012, China

**Keywords:** *Dendrobium huoshanense*, cultivation modes, nutritional constituents, gastroprotective effects

## Abstract

*Dendrobium huoshanense*, a rare and endangered medicinal orchid species endemic to Huoshan County, Anhui Province, faces a severe limit of wild resources. Its medicinal efficacy derives from unique chemical constituents, which vary significantly across cultivation modes. We systematically compared sensory properties and nutritional constituents (polysaccharides, flavonoids, alkaloids, bibenzyls, minerals, and free amino acids) between *D. huoshanense* in three cultivation modes—greenhouse cultivation, understory gravel cultivation, and wild-simulated cultivation. Additionally, the gastroprotective effects of wild-simulated cultivated *D. huoshanense* on gastric mucosal epithelial cells were evaluated. Results demonstrated that wild-simulated cultivation yielded significantly higher levels of nutritional constituents compared to gravel and greenhouse cultivation. Furthermore, wild-simulated cultivated *D. huoshanense* exhibited notable protective effects against gastric mucosal epithelial cell damage. This study not only elucidates the influence of cultivation modes on the chemical profile of *D. huoshanense* but also provides scientific evidence supporting its gastric protective properties, offering a foundation for its sustainable utilization and quality-oriented cultivation.

## 1. Introduction

*Dendrobium huoshanense* C. Z. Tang et S. J. Cheng, a premium medicinal and edible herb in Traditional Chinese Medicine (TCM), has been historically used for gastrointestinal and yin-nourishing therapies [1,2,3]. Documented since 200 AD in Shennong’s *Herbal Classic*, it remains a dual-purpose resource for therapeutics and functional foods [4]. Locally known as “Mihu” and traditionally consumed in soups or health supplements, stems of *D. huoshanense* exhibit a “sweet taste” and “slightly cold” nature, aligning with TCM principles for “heat-clearing and fluid-promoting” functions [5,6]. The unique medicinal and nutritive quality of *D. huoshanense* stems is fundamentally governed by its characteristic metabolites, primarily polysaccharides [7], flavonoids [8], bibenzyl derivatives [9], alkaloids [10], amino acids [11], trace elements [12], and phenolic compounds [13]. It has been proven that *D. huoshanense* polysaccharides are beneficial for cell growth and immune regulation [7]. Notably, stem-derived glucomannans and other polysaccharides confer the viscoelastic texture of *D. huoshanense* in food applications while concurrently mediating immunomodulation and mucosal protection [14,15], whereas stem-derived dendrobine-type alkaloids contribute to its characteristic bitterness [16]. Modern studies confirm *D. huoshanense*-specific bioactivities with food-compatible properties: anti-inflammatory [17], hypoglycemic [18], and gastrointestinal protective effects [19,20].

*D. huoshanense* is documented in Flora Reipublicae Popularis Sinicae as endemic to the southwestern Henan (Nanzhao) and Anhui (Huoshan) provinces. In the wild, this orchid grows as an epiphyte on cliffs along riverbanks and valleys at elevations of 200–1200 m. Due to its highly specific habitat requirements, it is now considered a Dabie Mountain endemic, with its core distribution centered in Huoshan County [21]. The species thrives in narrow ecological niches, primarily colonizing rocks near mountain streams in Lu’an. Its slow growth rate and low natural regeneration capacity, compounded by historical overharvesting, have driven wild populations to a critically endangered status [22]. To ease this pressure, large-scale propagation through seed-based tissue culture and subsequent field planting have expanded rapidly; by 2021 the total cultivated area had reached about 700 ha [23].

Three cultivation regimes dominate: greenhouse cultivation, understory gravel cultivation, and wild-simulated (habitat-mimicking) cultivation. For greenhouse cultivation, *D. huoshanense* is grown in a substrate of crushed stone mixed with shredded bark, and the greenhouse is equipped with misting and cooling systems to meet the plant’s requirements. Understory gravel cultivation is carried out on mountain slopes dominated by broad-leaved, coniferous, or mixed forest with stout trunks; the stand should be well ventilated and of moderate density. The substrate is composed of gravel, sawdust, pine bark, and moss. Wild-simulated cultivation is established in cliff-side rock crevices that offer suitable temperature, humidity, light, and proximity to water. Seedlings are transplanted into these crevices and left to develop naturally, with native mountain rock serving as the substrate. Both the understory gravel and wild-simulated systems are designed as ecological plantings that reproduce key features of the natural habitat—light intensity, air humidity, and substrate chemistry—to obtain product quality close to that of wild plants while maintaining ecosystem integrity and long-term resource sustainability. Research has shown that light, water, and temperature strongly influence secondary metabolite accumulation [24], and the harvest time not only affects the accumulation of secondary metabolites but also has additional independent effects [25,26]. Consequently, plants raised under the three cultivation regimes differ markedly in morphology, yield, chemical profile, and bioactivity. Previous studies have identified primary components in *D. huoshanense* under different cultivation modes [27] but have not systematically analyzed nutritional constituents.

This study systematically compared the compositional differences in *D. huoshanense* under three cultivation modes. This study aims to systematically compare the sensory traits and chemical profiles of *D. huoshanense* produced under three cultivation modes and to identify the mode that exhibits the greatest gastroprotective activity in ethanol-injured GES-1 cells.

## 2. Results

### 2.1. Odor Characteristics of D. huoshanense Under Different Cultivation Modes

**Figure 1:**
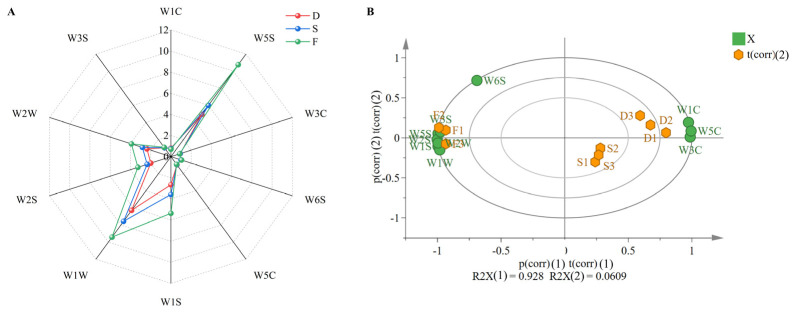
Electronic nose analysis of *D. huoshanense*. D: greenhouse cultivation, S: understory gravel cultivation, F: wild-simulated cultivation. (**A**) Radar chart of electronic nose responses, (**B**) biplot of principal component analysis electronic nose data.

Electronic nose analysis of *D. huoshanense* odor profiles revealed statistically significant differentiation among cultivation modes (greenhouse, understory gravel, and wild-simulated). Each cultivation mode was tested in triplicate, with the average sensor response values used to generate radar charts for comparative analysis. As shown in Figure 1A, the sensor responses followed the order greenhouse < understory gravel < wild-simulated cultivation. Significant differences were observed in five sensors (W1S, W1W, W2S, W2W, and W5S), which showed response values > 1, indicating these sensors detected the major volatile components contributing to the characteristic odor. In contrast, sensors W3C, W6S, W5C, W3S, and W1C exhibited negligible responses (<1). These results suggest that odor variations among cultivation modes primarily arise from differences in sulfur compounds, aromatic substances, nitrogen oxides, alkanes, and alkenes, with wild-simulated samples showing consistently higher responses in these five sensor categories. Principal component analysis (PCA) of the electronic nose data (Figure 1B) revealed clear separation among cultivation modes, confirming distinct odor characteristics. The first two principal components accounted for 98.9% of total variance (PC1: 92.8%; PC2: 6.1%), demonstrating their effectiveness in representing the original dataset. Additionally, the three cultivation patterns of *D. huoshanense* could also be distinguished, with the figure showing W1C, W5C, and W3C associated with PC1, and W6S associated with PC2. This PCA pattern strongly supports the reliability of the sensor response data in differentiating cultivation modes based on volatile compound profiles. The sensor characteristics are presented in Table S3.

### 2.2. Taste Profile Analysis of D. huoshanense Under Different Cultivation Modes

**Figure 2:**
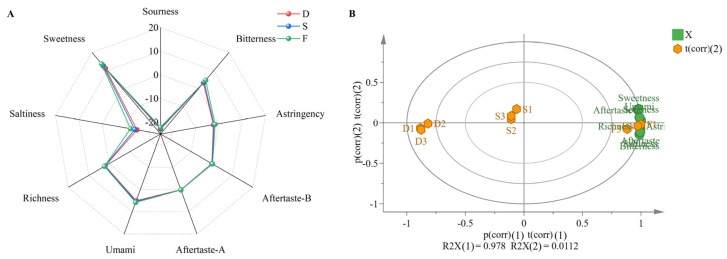
Electronic tongue analysis of *D. huoshanense*. D: greenhouse cultivation, S: understory gravel cultivation, F: wild-simulated cultivation. (**A**) Radar chart of taste attributes, (**B**) biplot of principal component analysis of electronic tongue data.

Electronic tongue analysis revealed significant taste differentiation among *D. huoshanense* in the cultivation modes. Under different cultivation modes, the sensors showed differences in responses to astringency aftertaste, bitterness aftertaste, astringency, richness, bitterness, and acidity, with significant differences observed in sweetness, umami, and saltiness sensors (Figure 2A). Nine sensor parameters were recorded with triplicate measurements per cultivation mode. One-way ANOVA with LSD post hoc testing revealed statistical differences (*p* < 0.05) in all nine sensor responses among the cultivation modes (Table 1), demonstrating the electronic tongue’s discriminative capacity. LSD results were alphabetically labeled (a, b, c), where shared letters indicate non-significant differences. PCA (Figure 2B) showed 98.9% cumulative variance explained (PC1: 97.8%; PC2: 1.1%), with distinct clustering of cultivation modes in the score plot, confirming significant taste profile differences.

### 2.3. Partial Least Squares Discriminant Analysis (PLS-DA) of D. huoshanense Under Cultivation Modes

**Figure 3:**
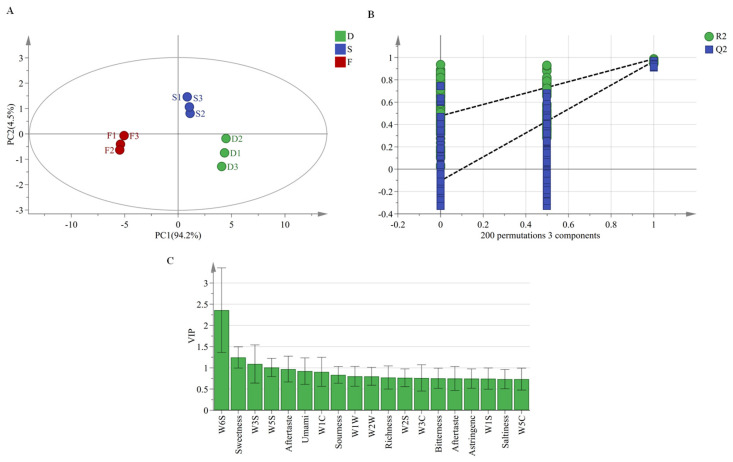
PLS-DA modeling analysis. D: greenhouse cultivation, S: understory gravel cultivation, F: wild-simulated cultivation. (**A**) PLS-DA score plot, (**B**) permutation test plot (*n* = 200), (**C**) variable importance in projection (VIP) plot.

PLS-DA [28], a supervised multivariate statistical approach, was applied to analyze flavor and taste characteristics of *D. huoshanense* under different cultivation modes. This method enhances model discrimination by reducing classification-independent variations while emphasizing group-specific patterns in principal components. The PLS-DA model (Figure 3A) showed variance explanation rates of 94.2% (PC1) and 4.5% (PC2), with a cumulative 98.7% contribution. Hotelling’s T^2^ test (95% confidence level) confirmed model reliability. High predictive ability was demonstrated by R^2^X (0.99), R^2^Y (0.984) and Q^2^ (0.958) values. Permutation testing (*n* = 200) validated model stability, with all permuted R^2^/Q^2^ values (Figure 3B) falling below original model values, indicating no overfitting. Variable importance in projection (VIP) analysis identified four key discriminators (VIP > 1.0): sensors W6S (hydrides), W3S (alkanes and aliphatics), and W5S (nitrogen oxides (NOx), and sweetness response (Figure 3C). These markers effectively differentiated three cultivation modes based on volatile and taste profiles.

### 2.4. Constituents of D. huoshanense Under Different Cultivation Modes

**Figure 4:**
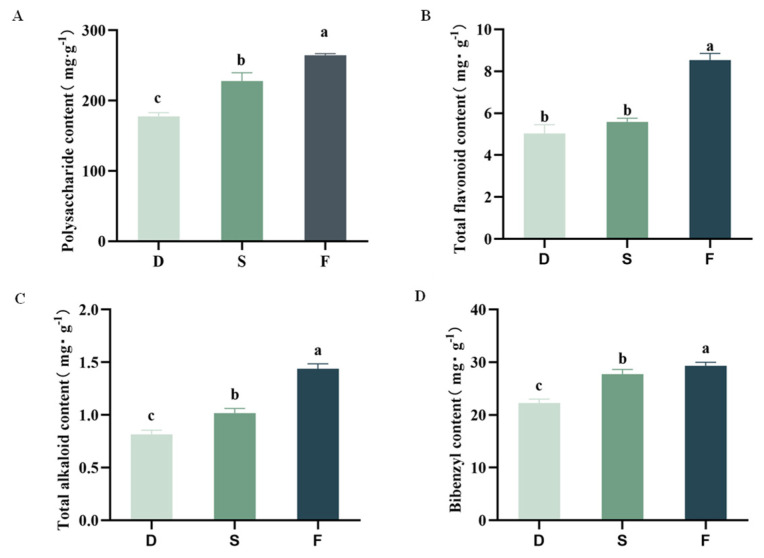
Effects of cultivation modes on *D. huoshanense* quality. D: greenhouse cultivation, S: understory gravel cultivation, F: wild-simulated cultivation. (**A**) Polysaccharide content, (**B**) total flavonoid content, (**C**) total alkaloid content, (**D**) bibenzyl content. Different lowercase letters (a, b, c) indicate significant differences at *p* ≤ 0.05 (Tukey’s HSD test).

As shown in Figure 4A–D, cultivation modes significantly influenced the compound contents of *D. huoshanense* (*p* < 0.05). Wild-simulated cultivation yielded the highest polysaccharide content (264.72 ± 2.01 mg·g^−1^), significantly surpassing greenhouse cultivation (177.70 ± 5.22 mg·g^−1^). A similar trend was observed for total flavonoids, with wild-simulated samples showing peak levels (8.54 ± 0.32 mg·g^−1^), though no significant difference existed between understory gravel and greenhouse modes. All three cultivation modes exhibited significant variations in total alkaloids and bibenzyls (*p* < 0.05). Wild-simulated *D. huoshanense* contained maximal concentrations of both total alkaloids (1.44 ± 0.05 mg·g^−1^) and bibenzyls (29.34 ± 0.7 mg·g^−1^), while greenhouse-grown specimens showed minimal values (0.82 ± 0.04 mg·g^−1^ and 22.29 ± 0.73 mg·g^−1^, respectively).

### 2.5. Trace Element Profiles in D. huoshanense Under Different Cultivation Modes

**Figure 5:**
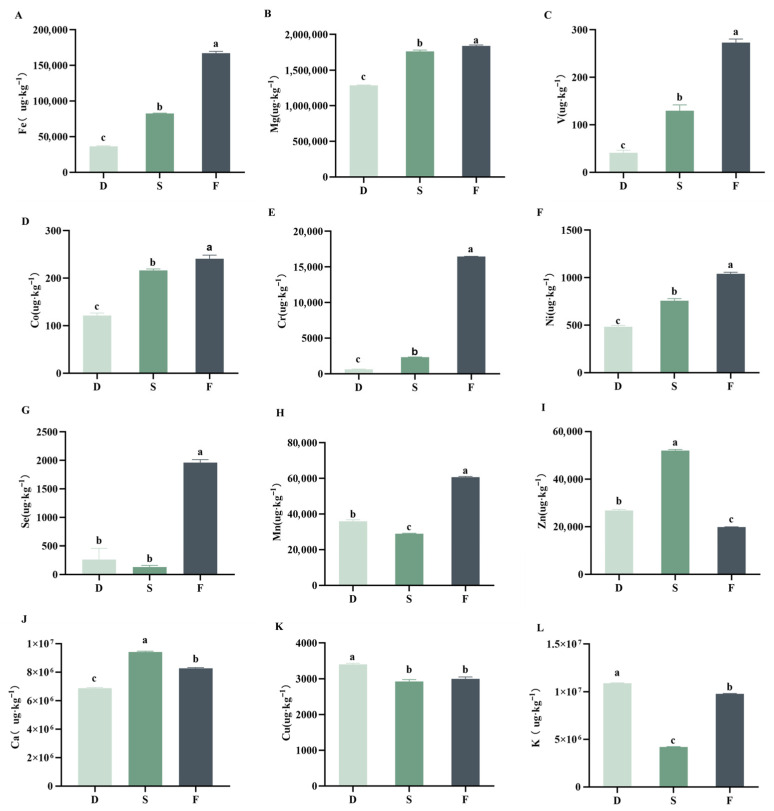
Trace element contents of *D. huoshanense* under different cultivation modes. Trace element concentrations are determined on a dry-weight basis (μg kg^−1^ DW). D: greenhouse cultivation, S: understory gravel cultivation, F: wild-simulated cultivation. (**A**) Fe content, (**B**) Mg content, (**C**) V content, (**D**) Co content, (**E**) Cr content, (**F**) Ni content, (**G**) Se content, (**H**) Mn content, (**I**) Zn content, (**J**) Ca content, (**K**) Cu content, (**L**) K content. Different letters (a, b, c) indicate statistically significant differences (*p* ≤ 0.05).

Trace elements, as essential micronutrients, are present in minute quantities in the human body and are indispensable for health, and they are usually absorbed in nutritional food. The standard curve of 12 trace elements is shown in Table S1. Figure 5A–L demonstrates distinct accumulation patterns of these elements in *D. huoshanense* across different cultivation modes. Wild-simulated cultivation yielded the highest iron (Fe) content, followed by rock-based understory cultivation, with greenhouse-grown specimens showing the lowest levels. Five elements (Mg, V, Co, Cr, and Ni) exhibited concentration trends identical to Fe. Selenium (Se) and manganese (Mn) displayed an inverse pattern—understory gravel samples contained the lowest quantities, while wild-simulated plants accumulated the most. Conversely, zinc (Zn) and calcium (Ca) concentrations were minimal in wild-simulated groups but peaked in understory gravel cultivation. For copper (Cu) and potassium (K), the highest content was found in greenhouse cultivation, followed by wild-simulated samples. These distinct profiles suggest wild-simulated cultivation enhances accumulation of essential elements (Mg/Se) required for the organisms’ well-being as they play crucial roles in anti-inflammatory metabolic pathways [29]. Iron not only indirectly affects anti-inflammatory effects by regulating immunity but also participates in oxygen transport as well as the synthesis of neurotransmitters such as dopamine and serotonin [30,31].

### 2.6. Free Amino Acid Profiles in D. huoshanense Under Different Cultivation Modes

*Dendrobium* species are rich in amino acids, particularly the essential ones that the human body cannot synthesize. Moreover, amino acids significantly shape the taste profile of *D. huoshanense*. Flavor-active amino acids were categorized into three groups: umami (glutamic, aspartic), sweet (glycine, alanine, serine, proline, histidine), and bitter types (isoleucine, leucine, valine, arginine, tyrosine, and tryptophan) [32,33,34]. As shown in Table 2, understory gravel cultivation produced the highest glycine and tyrosine levels among sweet amino acids, while greenhouse cultivation showed the lowest concentrations of them. The remaining four sweet amino acids (alanine, serine, proline, and histidine) peaked in wild-simulated samples, followed by understory gravel cultivation. Notably, the elevated sweet amino acids in wild-simulated cultivation directly contribute to the characteristic “sweetness” of premium *D. huoshanense*, aligning with traditional sensory evaluations that associate natural sweetness with superior medicinal quality [35]. For umami amino acids, wild-simulated plants accumulated maximal aspartic and glutamic acid contents, contrasting with minimal levels in greenhouse specimens. Bitter amino acids exhibited mode-specific patterns, with leucine peaking in understory gravel *D. huoshanense.* Wild-simulated cultivation significantly outperformed other modes in total flavor-active amino acids (sweet: 1028.20 ± 103.87 ng·mg^−1^; umami: 829.17 ± 92.35 ng·mg^−1^; bitter: 2359.72 ± 248.73 ng·mg^−1^). These findings align with the electronic tongue results, demonstrating that wild-simulated conditions enhance flavor amino acid accumulation in *D. huoshanense*.

To validate the applicability of the established method for amino acid determination, key parameters including linear range, linearity, limit of detection (LOD), and precision were systematically evaluated. The method was validated by assessing linearity, dynamic range, detection limits, and intra-day and inter-day relative standard deviations (RSDs). As shown in Table S2, all 13 amino acids exhibited good linearity within their respective concentration ranges, with correlation coefficients exceeding 0.99. Both intra-day and inter-day precision results demonstrated RSD values below 15%.

### 2.7. Protective Effects of D. huoshanense on Gastric Mucosal Epithelial Cell (GES-1) Injury

**Figure 6:**
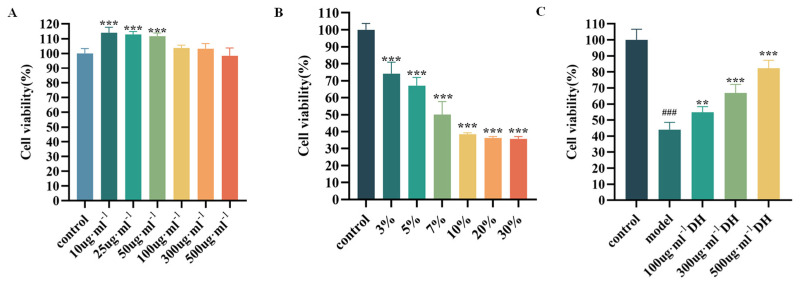
(**A**) Effects of different concentrations of *D. huoshanense* juice on cell viability *** *p* < 0.001 vs. control group. *X*-axis represents the concentration of *D. huoshanense* stem juice extract. (**B**) Effects of various ethanol concentrations on GES-1 cell viability. *X*-axis represents the concentration of ethanol. (**C**) Protective effects of *D. huoshanense* juice at different concentrations against ethanol-induced damage in GES-1 cells. The inflammation model is 7% ethanol. ^###^ *p* < 0.001 vs. control group; ** *p* < 0.01, *** *p* < 0.001 vs. model group.

The gastroprotective effects of *D. huoshanense* stem juice extract (DHJE) were systematically evaluated using ethanol-injured GES-1 cells. Initial screening identified 100–500 μg·mL^−1^ as optimal non-cytotoxic concentrations (Figure 6A), excluding lower doses (10–50 μg·mL^−1^) that altered basal viability (*p* < 0.001). Based on this result, the three concentrations of DHJE were selected as low (100 μg·mL^−1^), medium (300 μg·mL^−1^), and high (500 μg·mL^−1^) doses for subsequent experiments.

An ethanol-induced gastric injury was established by exposing GES-1 cells to a range of 3–30% ethanol concentrations, resulting in progressive viability reduction (74.15 ± 6.71%, 67.04 ± 4.86%, 50.08 ± 7.65%, 38.41 ± 0.85%, 36.29% ± 0.83, and 35.72 ± 1.39% survival rates, respectively; Figure 6B). The 7% ethanol concentration, which reduced viability by approximately 50.08 ± 7.65%, was chosen for subsequent 30 min exposure experiments for modeling consistent mucosal injury.

Pretreatment with DHJE for 2 h prior to 7% ethanol exposure significantly attenuated cellular injury on GES-1 (Figure 6C). While ethanol treatment alone markedly decreased viability versus controls (*p* < 0.001), all three DHJE dose groups showed statistically significant protection against ethanol-induced damage (*p* < 0.01).

**Figure 7:**
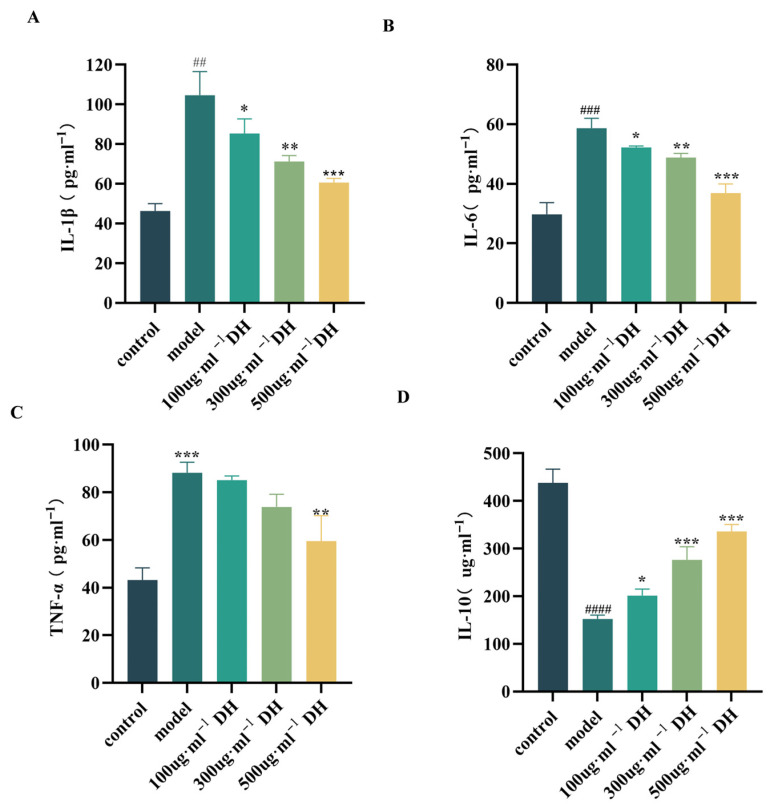
Effects of *D. huoshanense* on inflammatory cytokine levels in gastric mucosal epithelial cell injury. (**A**) IL-6 level, (**B**) IL-1β level, (**C**) TNF-α level, (**D**) IL-10 level. The inflammation model is 7% ethanol. ^####^ *p* < 0.0001, ^###^ *p* < 0.001, ^##^ *p* < 0.01 vs. control group; * *p* < 0.05, ** *p* < 0.01, *** *p* < 0.001 vs. model group.

Mechanistic studies revealed that ethanol exposure triggered characteristic inflammatory responses, significantly elevating pro-inflammatory cytokines (Interleukin-6 (IL-6), Interleukin-1β (IL-1β), and Tumor Necrosis Factor-α (TNF-α)) while suppressing anti-inflammatory Interleukin 10 (IL-10) (Figure 7A–D). DHJE treatment dose-dependently reversed these effects, reducing pro-inflammatory cytokines and enhancing the anti-inflammatory cytokine, indicating its dual mechanism of gastric mucosal protection through inflammatory pathway modulation.

## 3. Discussion

The accumulation of bioactive metabolites in medicinal plants, while fundamentally governed by genetic encoding, is dynamically modulated by environmental determinants. Most plants regulate the types and quantities of secondary metabolites according to the growth environment [36]. For habitat-specialized species like *D. huoshanense*, niche factors (light, humidity, and substrate) directly govern stress-inducible metabolite biosynthesis—notably polysaccharides, alkaloids, glycosides, and steroids [37]. In this study, by analyzing the correlation between component content and in vitro bioactivity, we obtained preliminary evaluation results of the quality of *D. huoshanense* under three cultivation patterns. Simulated wild cultivation environments have different humidity and greater temperature fluctuations, which may be key factors contributing to differences in taste [38]. The accumulation of trace elements may also have an effect, which could be related to the impact of soil microbial activity, soil texture, and pH on absorption and conversion efficiency under different ground management practices [39]. Minerals are extensively involved in the metabolism of carbohydrates, proteins, amino acids, vitamins, and other phytochemicals in crops, thereby affecting their composition [40,41]. The interactions among these components may lead to differences in the content of *D. huoshanense* under different cultivation patterns.

The compact and rock-adherent growth of *D. huoshanense* under wild-simulated cultivation results in short, fusiform stems with swollen internodes with a swollen, bulging middle. This unique growth on the rock represents an ecological adaptation that simultaneously enhances accumulation of chemical components such as polysaccharides and amino acids. The Carbon/Nutrient Balance (CNB) theory [42] further explains that when the impact of nutrient stress on plant growth is greater than that of photosynthesis, plants accumulate more carbon (C) and hydrogen (H), leading to an increase in the C/N ratio and enhancing the biosynthesis of carbon-based secondary metabolites such as phenolics and terpenes. This metabolic shift increases the production of volatile terpenoids, so that wild-simulated *D. huoshanense* exhibits a more intense volatile profile in electronic nose analysis. Based on the present findings, wild-simulated *D. huoshanense* exhibited markedly higher sweetness, umami, and bitterness scores. Considering the content of chemical components and flavor, taste profile is directly proportional to the contents of sweet, umami, and bitter amino acids, respectively, while its bitterness intensity also correlated positively with the total alkaloid level.

Wild-simulated cultivated *D. huoshanense* demonstrates significantly higher contents of polysaccharides, flavonoids, bibenzyls, and alkaloids compared to greenhouse- and understory gravel-cultivated ones. This metabolic enhancement results from two key environmental factors in its rocky habitats: intense ultraviolet (UV) radiation and reduced water availability. These findings align with established research confirming that both UV exposure and drought conditions promote secondary metabolite accumulation in medicinal plants [43]. Additionally, the performance of plants is closely related to their microbial communities. The cultivation substrate of *D. huoshanense* varies under different cultivation modes, and *D. huoshanense* is influenced by the microbial communities of the substrate. Substrates that mimic wild conditions may harbor high-quality microbial communities, which can increase the content of polysaccharides, flavonoids, alkaloids, and bibenzyl in *D. huoshanense* [44]. This type of wild-simulated cultivation environment is harsher, and compared to the other two cultivation modes, the leaves are smaller, the stems are thicker, and the roots are more developed. Such living conditions may increase the content of polysaccharides and alcohol-soluble extracts [45]. Wu et al. [46] conducted proteomics and phosphoproteomics in *D. huoshanense* between understory gravel cultivation and greenhouse cultivation. They also found significant changes in proteins related to photosynthesis, polysaccharide, and alkaloid biosynthesis pathways from the view of proteomics, which may explain the molecular mechanisms underlying the differences between various cultivations of *D. huoshanense.* Among free amino acids, proline and arginine serve as key biomarkers of abiotic stresses (e.g., drought or saline–alkali conditions). The environmental factors experienced by wild-simulated *D. huoshanense* promote significant accumulation of these stress-responsive amino acids, a finding corroborated by our results showing elevated total amino acid content under such cultivation conditions. Sun et al. [47] found that the nutritional quality index of trace elements in *D. huoshanense* is higher than the recommended intake. Therefore, *D. huoshanense* is rich in trace elements and is very important as a nutritional element. *D. huoshanense* naturally colonizes mineral-rich rocky substrates, absorbing abundant mineral elements that accumulate in the stems [47]. Consequently, wild-simulated plants exhibit significantly higher trace element contents (Fe, Mg, V, Co, Cr, and Ni) than either greenhouse- or gravel-cultivated under-forest specimens. Thus, the synergistic interplay of morphological adaptation, carbon reallocation, and geochemical uptake establishes wild-simulated cultivation as the optimal strategy for maximizing both medicinal and sensory quality parameters in *D. huoshanense.*

Preliminary studies on wild-simulated cultivation techniques and their influencing factors have been conducted in several *Dendrobium* species. Luo et al. [48] reported that total alkaloids and polysaccharides in wild-tree-simulated *Dendrobium officinale* were significantly higher than those in greenhouse, rock, or pot cultivation. Similarly, among the three cultivation modes compared here, wild-simulated *D. huoshanense* showed the highest levels of polysaccharides, flavonoids, total alkaloids, and amino acids, whereas greenhouse-grown samples had the lowest. Thus, wild-simulated cultivation yields *D. huoshanense* with higher active-ingredient content and superior quality, consistent with the findings of Yi et al. [22]. Hao et al. [49] demonstrated that neutral polysaccharides isolated from *D. huoshanense* effectively inhibit pro-inflammatory cytokine release from LPS-stimulated macrophages, exhibiting anti-inflammatory and immunomodulatory activities. Furthermore, we found here that the juice of wild-simulated *D. huoshanense* stems dose-dependently reduced pro-inflammatory cytokines and enhanced an anti-inflammatory cytokine, indicating its gastric mucosal protection due to its anti-inflammatory effect.

Gastric cancer remains a significant global health concern with persistently high incidence and mortality rates [50]. The disease progression follows a well-defined multistep cascade, beginning with chronic superficial gastritis and sequentially advancing through chronic atrophic gastritis, intestinal metaplasia, and dysplasia, before culminating in early and ultimately advanced gastric cancer [51,52]. This pathogenic trajectory underscores the critical importance of reversing gastric precancerous lesions as a fundamental strategy for gastric cancer prevention and management. TCM approaches have demonstrated particular efficacy in addressing these precancerous conditions, representing a distinct therapeutic advantage in our healthcare system. Our research advances this field by establishing wild-simulated *D. huoshanense* as a potent intervention, where maximal polysaccharide accumulation with co-accumulated bibenzyls and proline confer superior gastroprotection. Current research has established that *D. huoshanense* polysaccharides exhibit protective effects on the gastric mucosal barrier in ethanol-induced ulcer models [53]. Furthermore, various polysaccharides derived from the *Dendrobium* genus have been shown to dose-dependently mitigate ethanol-induced damage in human GES-1 models of acute mucosal injury [54,55]. Mechanistically validated by Liang et al. [56], polysaccharides in *D. officinale* (DOPs) modulate inflammatory responses through the NLRP3 inflammasome signaling pathway, effectively rebalancing pro- and anti-inflammatory cytokine ratios (notably reducing TNF-α and IL-1β levels) while attenuating colonic mucosal inflammation in acute colitis models. Our measurements revealed that wild-simulated *D. huoshanense* with higher polysaccharide content exerted gastric protection via anti-inflammatory mechanisms, evidenced by significant mitigation of mucosal injury. These results collectively position the juice of wild-simulated *D. huoshanense* stems as a comprehensive therapeutic and prevention agent against gastric injury and even carcinogenesis.

## 4. Materials and Methods

### 4.1. Sample Collection

Fresh *D. huoshanense* specimens were purchased from the Huoshan production base of Jiuxianzun Huoshan Dendrobium Co., Ltd., in Lu’an, Anhui Province, China. Three-year-old seedlings of *D. huoshanense* C. Z. Tang et S. J. Cheng with uniform, vigorous growth were collected from the cultivation base in Taipingfan Town, Huoshan County, Anhui Province, China. The fresh specimens were collected in April, and the stems were used as experimental material. The plants were cultivated using three modes: greenhouse cultivation, understory gravel cultivation, and wild-simulated cultivation. Taxonomic identification was performed by Professor Yu Nianjun of the Anhui University of Chinese Medicine, confirming the specimens as *D. huoshanense*. Voucher specimens of plants were deposited at the Herbarium Center, Anhui University of Chinese Medicine, Hefei, China (Daiyin Peng, pengdy@ahtcm.edu.cn, Voucher Nos. 20221104, 20221105, and 20221106). The human gastric mucosal epithelial cell line (GES-1) was purchased from Saibai Kang Biotechnology Co., Ltd. (Shanghai, China). Samples were processed by first thoroughly washing them to remove roots and leaves, followed by drying in an oven at 60 °C until constant weight. The dried material was then ground into fine powder and passed through a 50-mesh sieve to obtain homogeneous sample particles suitable for subsequent analysis. The sample was legally collected in accordance with local legislation. No permission or license was required in this study. Each measurement and analysis were performed using three sample replicates.

### 4.2. Chemicals

D-glucose (BS099, ≥99%, Beijing Labgic Technology Co., Ltd., Beijing, China); rutin (B28707, ≥98%, Shanghai Yuanye Bio-Technology Co., Ltd., Shanghai, China); dendrobine (PS020540, ≥98%, Chengdu Push Bio-Technology Co., Ltd., Chengdu, China); dendrophenol (B21163, ≥95%, Shanghai Yuanye Bio-Technology Co., Ltd., Shanghai, China); 1× pbs buffer (Labgic Technology Co., Ltd., Beijing, China); phenol, sodium hydroxide, aluminum nitrate nonahydrate, sodium nitrite, potassium biphthalate, Folin–Ciocalteu’s phenol reagent, and casein (Macklin Biochemical Co., Ltd., Shanghai, China); dendrophenol and rutin (Yuanye Bio-Technology Co., Ltd., Shanghai, China); ammonia solution (Suyi Chemical Reagent Co., Ltd., Shanghai, China); sodium carbonate anhydrous and bromocresol green as indicator (Aladdin Biochemical Technology Co., Ltd., Shanghai, China); RPMI 1640 medium (Gibco, Thermo Fisher Scientific, Waltham, MA, USA); and fetal bovine serum (FBS) (Kangyuan Biotechnology Co., Ltd., Mingguang, Anhui, China).

### 4.3. Electronic Nose Analysis

A total of 2 g of *D. huoshanense* was accurately weighed and put in a 50 mL centrifuge tube, sealed with sealing film, and equilibrated for 24 h before analysis. Volatile compound profiling was performed with an electronic nose system (AIRSENSE Analytics GmbH, Schwerin, Germany) under fixed conditions: sampling frequency 1 Hz, sensor purge 90 s, baseline correction 5 s, and carrier-gas flow 300 mL min^−1^. Each acquisition lasted 120 s and was repeated in triplicate. Sensor response data at the 90 s time point were selected for statistical evaluation based on signal stability criteria.

### 4.4. Electronic Tongue Analysis

An accurately weighed 0.5 g of *D. huoshanense* was transferred to a 50 mL centrifuge tube and mixed with 50 mL ultrapure water. After standing at room temperature for 1 h, the slurry was subjected to ultrasonic-assisted extraction for 2 h. The mixture was then centrifuged at 4000× *g* for 15 min, and the supernatant was collected. The residue was re-extracted with a fresh 50 mL aliquot of ultrapure water under sonication for 1 h and centrifuged under identical conditions. The two supernatants were combined to give the final test solution. Taste attributes—bitterness, astringency, sourness, saltiness, umami, and sweetness—were evaluated with an electronic tongue (Intelligent Sensor Technology, Inc., Atsugi, Japan) comprising a main unit, disposable lipid membrane sensors, and control software. Sensors were activated and calibrated prior to use. Each measurement cycle began with a 90 s rinse in cleaning solution, followed by two consecutive 120 s rinses in reference solutions, and a 30 s stabilization step at the baseline position. Four replicate cycles were run; the first was discarded and the mean of the remaining three was used for analysis.

### 4.5. Determination of Polysaccharide Content of D. huoshanense

Following a modified protocol adapted from Yang et al. [57], 0.4 g of sample powder was accurately weighed and extracted with 200 mL deionized water under reflux at 100 °C for 2 h. After cooling to room temperature, the extract was quantitatively transferred to a 250 mL volumetric flask. The container was rinsed several times with small amounts of deionized water, and the washings were combined and brought to volume. The solution was filtered through filter paper. An aliquot of the filtrate was mixed with 10 mL absolute ethanol in a 15 mL centrifuge tube, precipitated at 4 °C for 1 h. The mixture was centrifuged at 4000× *g* for 20 min at 4 °C, and the supernatant was discarded. The precipitate was washed twice with 8 mL of 80% (*v*/*v*) ethanol solution, followed by centrifugation under identical conditions. The final precipitate was dissolved in hot water and quantitatively transferred to a 25 mL volumetric flask, then brought to volume after cooling to obtain the polysaccharide test solution. Absorbance measurements were performed at 490 nm using a UV–Vis spectrophotometer (Agilent Technologies Co., Ltd., Shanghai, China). A standard curve was established using glucose reference material with the regression equation y = 10.51x − 0.0113 (R^2^ = 0.9945), where y represents absorbance and x denotes glucose concentration (mg·mL^−1^). Each sample was analyzed in triplicate, and the mean value was used for polysaccharide content calculation.

### 4.6. Determination of Total Flavonoid Content of D. huoshanense

Following a modified protocol adapted from Li et al. [58], 2.0 g of homogenized powder was subjected to ultrasonic-assisted extraction using 25 mL of 70% ethanol in sealed conical flasks. After cooling to room temperature, the extract was filtered through quantitative filter paper. The residue was washed three times with small aliquots of 70% ethanol, and the combined filtrates were diluted to 50 mL with additional 70% ethanol in a volumetric flask to obtain the test solution. Total flavonoid content was determined using a UV–Vis spectrophotometer at 510 nm. A standard calibration curve was constructed using rutin as the reference standard. The regression equation was y = 11.152x + 0.009 (R^2^ = 0.9986), where x represents rutin concentration (mg·mL^−1^) and y corresponds to absorbance. Triplicate determinations per biological replicate (*n* = 3) were performed.

### 4.7. Determination of Total Alkaloid Content of D. huoshanense

Precisely 0.5 g of *D. huoshanense* powder was weighed into a 100 mL round-bottom flask and subjected to alkalization treatment with ammonia solution in a sealed system for 30 min. Following the addition of chloroform, continuous extraction was performed using Soxhlet apparatus with water bath reflux for 2 h. After cooling to room temperature, the extract was reweighed and any chloroform loss was compensated before filtration. The filtrate was then processed through a two-step dilution protocol: initially diluted to 10 mL in a volumetric flask, followed by transfer of an aliquot to a 25 mL volumetric flask after thorough mixing. The final test solution was prepared by diluting to volume with distilled water and homogenizing through vigorous shaking. Absorbance measurements were performed at 620 nm using a UV–Vis spectrophotometer. A calibration curve was established using the reference standard of dendrobine, resulting in the regression equation Y = 0.0515x + 0.0222 (R^2^ = 0.9963), where x represents the concentration of dendrobine (μg·mL^−1^) and Y corresponds to the absorbance. Triplicate measurements were conducted for each sample, and the mean values were used to calculate the total alkaloid content.

### 4.8. Determination of Bibenzyl Content of D. huoshanense

For bibenzyl quantification, 0.5 g of *D. huoshanense* powder was accurately weighed into a 100 mL round-bottom flask. The sample was extracted with 70% (*v*/*v*) ethanol under sonication for 1 h. The resulting extract was filtered through quantitative-grade filter paper to obtain the filtrate. Casein was added to the filtrate, and the mixture was incubated in a water bath at 30 °C with constant shaking for 1 h. Upon reaction completion, the mixture was vacuum-filtered, and the filtrate was evaporated to complete dryness under reduced pressure. The residue was reconstituted in ultrapure water and quantitatively transferred to a 100 mL volumetric flask, which was then brought to volume with water to prepare the test solution. A calibration curve was established using dendrophenol reference standard by plotting the absorbance values of the chromogenic reaction measured at 760 nm against the corresponding concentrations. The calibration curve yielded the regression equation y = 5.0864x − 0.018 (R^2^ = 0.9966), where x represents dendrophenol concentration (mg·mL^−1^) and y corresponds to the absorbance value.

### 4.9. Trace Element Analysis in D. huoshanense

Trace element quantification employed microwave-assisted acid digestion followed by inductively coupled plasma mass spectrometry (ICP-MS) analysis. Precisely 0.5000 ± 0.0002 g powder of *D. huoshanense* powder was weighed. The samples were initially moistened with 1–2 mL of ultrapure water before adding 10 mL of concentrated nitric acid. After securely sealing the vessels, they were loaded into the microwave digestion system. The digestion protocol began with a 30 min pre-digestion at 120 °C, followed by a three-stage microwave program, first ramping to 130 °C in 5 min (3 min hold), then increasing to 150 °C over 3 min (10 min hold), and finally reaching 180 °C in 3 min (30 min digestion period). Cooled digests (60 °C) were diluted to 25 mL with 5% HNO_3_ (*v*/*v*). Elemental analysis was conducted using an Agilent 7800 ICP-MS system (Agilent Technologies, Santa Clara, CA, USA), where target elements and internal standards were simultaneously measured. Quantification was performed by comparing the obtained signal intensities against matrix-matched calibration curves.

### 4.10. Free Amino Acid Quantification

For free amino acid analysis, 15 mg of *D. huoshanense* sample was accurately weighed into a 1.5 mL microcentrifuge tube. The sample was sequentially treated with 141 μL of ultrapure water and 100 μL of 0.15% sodium deoxycholate (DOC) solution, followed by the addition of 4 μL of a mixed internal standard solution (containing L-lysine-d4, L-tryptophan-d5, and L-glutamine-d4, each at a concentration of 100 μg·mL^−1^). The mixture was then subjected to ultrasonic extraction for 10 min at 5 °C (40 kHz). Protein precipitation was achieved by adding 5 μL of 10 M trichloroacetic acid for incubation. The supernatant was diluted with 375 μL of ultrapure water and filtered through a 0.2 μm PTFE. Quantitative analysis was performed using an ultra-high-performance liquid chromatography–tandem mass spectrometry (UHPLC-MS/MS) system (AB SCIEX, Shanghai, China). Chromatographic separation was performed on an AdvanceBio MS Spent Media column (2.1 × 50 mm, 2.7 μm particle size) maintained at 40 °C, using an injection volume of 1 μL. The binary mobile phase system consisted of (A) an aqueous solution containing 0.1% formic acid and 10 mM ammonium formate in water (95:5, *v*/*v*) and (B) an organic solution containing 0.1% formic acid and 10 mM ammonium formate in acetonitrile (95:5, *v*/*v*). Mass spectrometric analysis was conducted using a SCIEX QTRAP 6500+ system operating in both positive and negative ionization modes. The optimized mass spectrometric parameters were as follows: curtain gas (CUR) 35 psi, collision gas (CAD) medium, ion spray voltage (IS) ±5500 V (positive/negative mode), source temperature 550 °C, and both ion source gases (GS1 and GS2) 50 psi. The analytical method employed scheduled multiple reaction monitoring (MRM) with basic parameter settings and a 120 s detection window.

### 4.11. Protective Effects of D. huoshanense on GES-1 Cells Against Ethanol-Induced Damage

This study investigated the cytoprotective effects of the wild-simulated juice of stems of *D. huoshanense* on ethanol-induced gastric mucosal epithelial cell damage through a systematic experimental approach. The stems of wild-simulated *D. huoshanense* were mixed with ultrapure water at a solid-to-liquid ratio of 1:20 and processed in a juicer. The resulting slurry was first pre-filtered through gauze, and the filtrate was centrifuged to collect the supernatant, which was then concentrated by rotary evaporation until the extract reached a density of 1.2 g mL^−1^ and subsequently stored at −80 °C until use.

Initially, the safety profile and optimal working concentrations of the extract were determined by incubating GES-1 cells with graded concentrations (10, 25, 50, 100, 300, and 500 μg mL^−1^) followed by CCK-8 viability assessment. Parallel experiments established an in vitro gastric mucosal injury model using an ethanol gradient (3–30%), with cell viability measurements identifying the most effective damage-inducing concentration. Subsequent experiments employed a three-group design (normal control, ethanol model, and treatment groups). Pretreatment with the validated safe concentrations of extract preceded ethanol exposure in the treatment group, and CCK-8 assays quantified the protective effects on cell viability. To elucidate the underlying mechanisms, cytokine profiling was performed on supernatants collected from 6-well plate experiments. Enzyme-Linked Immunosorbent Assay (ELISA) quantification of inflammatory mediators IL-1β, IL-6, IL-10, and TNF-α followed the manufacturer’s protocols, with standard curve-based calculations determining cytokine concentrations.

### 4.12. Statistical Analysis

All experimental data were analyzed using IBM SPSS Statistics 27, SIMCA 14.1, and Origin 2021 software, with results expressed as mean ± standard deviation (Mean ± SD). Data visualization was performed using GraphPad Prism 9.5.0. For intergroup comparisons, one-way analysis of variance (ANOVA) was employed, with a *p*-value ≤ 0.05 considered statistically significant.

## 5. Conclusions

Among the three cultivation modes evaluated, wild-simulated *D. huoshanense* demonstrated significantly higher contents of most nutritive components including polysaccharides, total flavonoids, total alkaloids, bibenzyl compounds, amino acids, and trace elements, along with distinct organoleptic characteristics in terms of aroma and taste profiles. These findings indicate that wild-simulated cultivation yields *D. huoshanense* with superior medicinal quality. Furthermore, our study provides additional evidence for the gastric mucosal protective effects of *D. huoshanense* juice under wild-simulated cultivation, as demonstrated by its ability to mitigate cellular damage and maintain epithelial integrity.

## Figures and Tables

**Table 1:** Taste potential difference response values of electronic tongue sensors for *D. huoshanense* under different cultivation modes.

	Greenhouse Cultivation	Understory Gravel Cultivation	Wild-Simulated Cultivation	F	Statistical Significance
Sourness	−22.953 ± 0.025 c	−22.587 ± 0.021 b	−22.133 ± 0.040 a	562.420	0.001
Bitterness	3.167 ± 0.021 c	3.620 ± 0.036 b	4.650 ± 0.026 a	2137.000	0.001
Astringency	−2.347 ± 0.021 c	−2.063 ± 0.015 b	−1.603 ± 0.060 a	294.566	0.001
Aftertaste-B	0.030 ± 0.010 c	0.143 ± 0.015 b	0.337 ± 0.015 a	381.882	0.001
Aftertaste-A	0.083 ± 0.015 c	0.137 ± 0.015 b	0.190 ± 0.010 a	45.176	0.001
Umami	4.993 ± 0.061 c	5.367 ± 0.049 b	5.753 ± 0.030 a	183.061	0.001
Richness	2.000 ± 0.000 c	2.130 ± 0.010 b	2.337 ± 0.047 a	111.186	0.001
Saltness	−14.897 ± 0.070 c	−13.353 ± 0.118 b	−12.127 ± 0.023 a	915.345	0.001
Sweetness	11.187 ± 0.021 c	12.667 ± 0.055 b	13.677 ± 0.040 a	2767.824	0.001

Note: Different letters (a, b, c) indicate statistically significant differences (*p* ≤ 0.05).

**Table 2:** Contents of flavor-active amino acids in *D. huoshanense* under different cultivation modes.

	Cultivation Mode		D	S	F
Free Amino Acids			ng·mg^−1^		
Sweet amino acids		Alanine	191.52 ± 23.07	247.30 ± 3.82	299.89 ± 15.30
		Glycine	51.57 ± 16.93	82.86 ± 27.14	76.66 ± 14.57
		Histidine	183.53 ± 40.70	193.30 ± 29.07	295.43 ± 59.42
		Proline	117.97 ± 11.48	195.59 ± 1.65	225.50 ± 9.53
		Serine	78.91 ± 10.73	126.29 ± 35.03	130.71 ± 9.52
		Total	623.51 ± 97.56 c	845.34 ± 23.35 b	1028.20 ± 103.87 a
Umami amino acids		Aspartic acid	89.49 ± 9.40	99.54 ± 6.43	213.58 ± 11.03
		Glutamic acid	56.89 ± 3.81	158.47 ± 35.30	90.24 ± 3.51
		Lysine	347.46 ± 27.21	405.03 ± 22.16	525.33 ± 81.95
		Total	493.84 ± 32.88 c	663.04 ± 53.32 b	829.17 ± 92.35 a
Bitter amino acids		Isoleucine	83.35 ± 12.03	207.74 ± 3.62	191.08 ± 8.56
		Leucine	167.79 ± 17.15	250.40 ± 2.59	279.52 ± 21.79
		Valine	89.22 ± 9.72	153.92 ± 1.68	153.27 ± 8.92
		Arginine	553.890 ± 152.36	655.20 ± 71.25	1519.98 ± 204.02
		Tyrosine	86.84 ± 9.19	121.97 ± 3.00	153.15 ± 10.12
		Tryptophan	30.99 ± 3.89	36.59 ± 1.09	62.70 ± 8.21
		Total	1012.08 ± 203.58 c	1425.83 ± 66.72 b	2359.72 ± 248.73 a

D: greenhouse cultivation, S: understory gravel cultivation, F: wild-simulated cultivation. Different letters (a, b, c) indicate statistically significant differences (*p* ≤ 0.05).

## Data Availability

The data presented in this study are available on request from the corresponding author. The data are not publicly available due to privacy.

## Supplementary Materials

The following supporting information can be downloaded at https://www.mdpi.com/article/10.3390/plants14243718/s1, Table S1: Standard equation of trace elements; Table S2: Methodological investigation. Table S3: Sensitive substance type of each sensor.
