# Supplementary material for: Quality Evaluation of Dendrobium huoshanense Under Different Cultivation Modes and Its Protective Effect on Ethanol-Induced Injury in GES-1 Cells
Source: Plants (Basel). 2025 Dec 5;14(24):3718. doi: 10.3390/plants14243718 (PMC12737215; doi:10.3390/plants14243718)
Supplement: Supplementary file 1 [file plants-14-03718-s001.zip › Supplementary Materials.pdf]

Quality Evaluation of *Dendrobium huoshanense* under Different Cultivation Modes and Its Protective Effect on ethanol-induced injury in GES-1 cells-Supplementary Material

Table S1 Standard equation of trace elements

Trace Element	Curvilinear Equation	R^2
Fe	Y=0.0037x+0.0013	0.9996
Mg	Y=2.1444E-0.004x+3.6547E-0.005	1.0000
V	Y=0.003x+1.5457E-0.005	0.9996
Co	Y=0.0078x+3.7048E-0.005	0.9998
Cr	Y=0.0043x+3.2425E-0.004	0.9997
Ni	Y=0.0023x+9.0445E-0.004	0.9998
Se	Y=2.2076E-0.005x+0.0000E+0	0.9995
Mn	Y=0.0019x+9.8687E-0.005	0.9998
Zn	Y=8.7242E-0.004x+2.8397E-0.004	0.9998
Ca	Y=4.5924E-006x+1.5595E-0.005	0.9998
Cu	Y=0.0062x+0.0032	0.9996
K	Y=7.8443E-0.005x+0.004	0.9999

Table S2 Methodological Investigation.

Amino acids	Linear Range ($ng·mL^{-1}$)	Linear Equation	R^2	LOD （$ng·ml^{-1}$）	inter-day variability（%）	intra-day variability（%）
Alanine	6.4-40000	Y=269x-301	0.9965	6.4	3.1	8.2
Arginine	0.64-40000	Y=2997x+29742	0.9955	0.64	3.3	10.6
Aspartic acid	3.2-20000	Y=183x+6041	0.9923	3.2	7.7	6.9
Glutamic acid	3.2-20000	Y=458x+3300	0.9983	3.2	8.0	6.9
Glycine	32-150000	Y=19x+945	0.9913	32	3.0	8.5
Histidine	6.4-40000	Y=5085x+1	0.9989	6.4	2.2	7.0
Isoleucine	0.32-20000	Y=763x+3350	0.9981	0.32	12.8	10.5
Leucine	3.84-24000	Y=210x+1289	0.9969	3.84	3.8	10.5
Lysine	3.2-20000	Y=1.28x+0.17	0.9941	3.2	4.1	8.3
Proline	3.2-20000	Y=14208x+24277	0.9912	0.2	2.3	7.9
Serine	3.2-20000	Y=328x+889	0.9995	2.06	4.9	3.9
Tyrosine	3.2-20000	Y=1212x+2122	0.9987	3.2	6.4	6.3
Valine	3.2-20000	Y=3826x+10465	0.9993	3.2	1.5	5.5
Tryptophan	0.96-6000	Y=5468x+2568	0.9994	0.996	4.9	3.9

Table S3 Sensitive substance type of each sensor.

Sensor ID	Sensor Name	Target Substances
1	W1C	Aromatic compounds
2	W5S	Nitrogen oxides (NO_x)
3	W3C	Ammonia & aromatic compounds
4	W6S	Hydrides
5	W5C	Alkenes, aromatics, polar molecules
6	W1S	Alkanes

7	W1W	Sulfur compounds
8	W2S	Alcohols & partial aromatics
9	W2W	Aromatic sulfur compounds
10	W3S	Alkanes & aliphatics
